# China suboptimal health cohort study: rationale, design and baseline characteristics

**DOI:** 10.1186/s12967-016-1046-y

**Published:** 2016-10-13

**Authors:** Youxin Wang, Siqi Ge, Yuxiang Yan, Anxin Wang, Zhongyao Zhao, Xinwei Yu, Jing Qiu, Mohamed Ali Alzain, Hao Wang, Honghong Fang, Qing Gao, Manshu Song, Jie Zhang, Yong Zhou, Wei Wang

**Affiliations:** 1 Beijing Key Laboratory of Clinical Epidemiology, School of Public Health, Capital Medical University, Beijing, 100069 China; 2 Global Health and Genomics, School of Medical and Health Sciences, Edith Cowan University, Perth, 6027 Australia; 3 Department of Neurology, Beijing Tiantan Hospital, Capital Medical University, Beijing, 100050 China; 4 School of Public Health, Ningxia Medical University, Yinchuan, 750021 China; 5 Beijing Institute of Heart, Lung and Blood Vessel Diseases, Beijing Anzhen Hospital, Capital Medical University, Beijing, 100029 China; 6 Department of Neurology, Beijing Institute of Heart, Lung and Blood Vessel Diseases, Beijing Anzhen Hospital, Capital Medical University, Beijing, 100027 China

**Keywords:** Suboptimal health status (SHS), Non-communicable chronic disease (NCD), Cardiovascular events, Cerebrovascular events, Cohort study

## Abstract

**Background:**

Suboptimal health status (SHS) is a physical state between health and disease, characterized by the perception of health complaints, general weakness, chronic fatigue and low energy levels. SHS is proposed by the ancient concept of traditional Chinese medicine (TCM) from the perspective of preservative, predictive and personalized (precision) medicine. We previously created the suboptimal health status questionnaire 25 (SHSQ-25), a novel instrument to measure SHS, validated in various populations. SHSQ-25 thus affords a window of opportunity for early detection and intervention, contributing to the reduction of chronic disease burdens.

**Methods/design:**

To investigate the causative effect of SHS in non-communicable chronic diseases (NCD), we initiated the China suboptimal health cohort study (COACS), a longitudinal study starting from 2013. Phase I of the study involved a cross-sectional survey aimed at identifying the risk/protective factors associated with SHS; and Phase II: a longitudinal yearly follow-up study investigating how SHS contributes to the incidence and pattern of NCD.

**Results:**

(1) Cross-sectional survey: in total, 4313 participants (53.8 % women) aged from 18 to 65 years were included in the cohort. The prevalence of SHS was 9.0 % using SHS score of 35 as threshold. Women showed a significantly higher prevalence of SHS (10.6 % in the female vs. 7.2 % in the male, *P* < 0.001). Risk factors for chronic diseases such as socioeconomic status, marital status, highest education completed, physical activity, salt intake, blood pressure and triglycerides differed significantly between subjects of SHS (SHS score ≥35) and those of ideal health (SHS score <35). (2) Follow up: the primary and secondary outcomes will be monitored from 2015 to 2024.

**Conclusions:**

The sex-specific difference in prevalence of SHS might partly explain the gender difference of incidence of certain chronic diseases. The COACS will enable a thorough characterization of SHS and establish a cohort that will be used for longitudinal analyses of the interaction between the genetic, lifestyle and environmental factors that contribute to the onset and etiology of targeted chronic diseases. The study together with the designed prospective cohort provides a chance to characterize and evaluate the effect of SHS systemically, and it thus generates an unprecedented opportunity for the early detection and prevention of chronic disease.

## Background

Major chronic diseases such as hypertension, heart disease, stroke, cancer, chronic obstructive pulmonary disease and diabetes caused an estimated 35 million deaths in 2005, 80 % of which occurred in low and middle income countries such as China [1–3]. Mortality rates of non-communicable chronic diseases (NCD) have been declining in most western countries, but NCD are increasing in China as a result of adverse changes such as lifestyle, environmental pollution, diet and tobacco use [4–6]. In the past 30 years, China has experienced dramatic transformations in social and economic conditions, and these changes will continue to increase the incidences of major chronic diseases [7]. From 1990 to 2010, the proportion of people living in urban cities in China increased from 26 to 50 % [8, 9]. It is expected that urbanization in China will reach 60 % by 2020, according to the official forecast [10]. The rapid environmental changes accompanied with urbanization lead to the increasing prevalence of the major risk factors for NCD, including work stress, physical inactivity, unhealthy diet, and tobacco use; therefore, as a result, the prevalence of NCD will continue to increase [7].

China is a country with 5000 years of civilization, and traditional Chinese medicine (TCM) is one of the prestigious medical heritages in the world, with over 2 millennia of clinical practices [8, 9]. Unfortunately, some of the TCM conceptions have not been recognized internationally due to the lack of systemic evidenced supports [10]. Suboptimal health status (SHS) is such an example. SHS is a physical state between health and disease, characterized by the perception of health complaints, general weakness, chronic fatigue and low energy levels [11]. We have also developed a tool to measure SHS. Our suboptimal health status questionnaire-25 (SHSQ-25) assesses five components of health [11, 12]. To date, the SHSQ-25 as a self-reported survey tool has been validated in various populations, including European ethic group [12–16], and currently SHSQ-25 has also being applied to a real life community-based health survey in Ghana, western Africa. SHS thus has been recognized internationally and it works a novel tool for the early detection of chronic disease [12–16]. We also found SHS to be associated with cardiovascular risk factors and may contribute to the development of cardiovascular disease. SHS has also been reported to be associated with chronic psychosocial stress [14–16] and poor lifestyle factors [17, 18].

Studies to improve early detection and intervention of NCD will become increasingly important, and the availability of reliable biomarkers for these diseases will be essential. Specific biomarkers, such as plasma glycome or serum peptidome, are believed to represent an ‘intermediate phenotype’ in the etiology of adult-onset diseases [19, 20]. Therefore, these profiles might hold the key in understanding the underlying biological mechanisms that create SHS. The SHSQ-25 promises to be a window of opportunity for early detection and intervention to reduce chronic disease burden [15]. The inclusion of the “objective” biomarkers and the subjective “SHS” assessment into population studies is therefore believed to be timely in improving chronic disease control and in strengthening opportunities for chronic disease prevention.

Although case-control studies may be sufficient for the investigation of potential impacts of genetic or environmental factors, large community-based prospective cohort studies are essential for the unbiased assessment of the relevance of both environmental and genetic factors, and their interactions. There have already been several prospective studies of major chronic diseases in China [21–25]. However, there are limitations, such as small sample size [21–23], lack of biological samples to measure biomarkers [24, 25], or limited definite information on environmental exposures and outcome of health status measures [21, 24, 25]. Moreover, the association between SHS and major chronic diseases in China is still poorly understood, and there is still substantial uncertainty about the present and future relevance to population morbidity and mortality of many common risk factors [25].

The China suboptimal health cohort study (COACS) uses a multidisciplinary approach to understand the impact of SHS on chronic diseases. The strategy is to study a moderately large cohort intensively, collecting data from a wide range of measures including physical function, cognition, medical history and SHS, as well as biological samples annually. This will assist in deciphering the important relationships between disease process and risk factors within individuals. The study aims to establish a cohort for investigators to comprehensively understand the potential significance of SHS combined with profiling of dynamic biomarkers for NCD pathogenesis. The COACS provides a platform from which early intervention strategies can be implemented and evaluated.

The COACS is designed based on the following hypothesis: the combination of genetic background, proofing of dynamic biomarkers and environmental exposures, in parallel with the application of the subjective health metrics (SHSQ-25) will contribute to risk stratification of chronic diseases, and serve as prognostic indicators for preventative treatment and interventions of chronic diseases.

## Methods/design

### Study design and participants

The COACS Study is a community-based, prospective study, to investigate how suboptimal health status contributes to the incidence of NCD in Chinese adults. The study has two phases, a cross-sectional survey, followed by a longitudinal study. The participants were recruited from *Caofeidian* district, *Tangshan* city, in northern China. *Caofeidian* district is located in the south of the *Tangshan* city and near the *Bohai* sea, with an area of 1944 km^2^ and a population of 268.7 thousand (According to 2012 China census), from September 2013 to June 2014. *Tangshan* is a large, modern industrial city located in the central section of the circum-*Bohai* region, where it adjoins two mega cities: *Beijing* and *Tianjin* (Table 1).

**Table 1:** Testing program in the China suboptimal health cohort study

Test	Components
Specimen collection	Fasting blood sample
Anthropometry	Height, weight, ankle-branchial index, waist and hip circumference
Participant break	Refreshment break with food provided
Cardiovascular	12 lead ECG, vascular profiling (blood pressures, pulse wave velocity), transcranial doppler, carotid artery sonography
Respiratory	Obstructive spirometry
Skeleton	Bone density examination
Gynecology (female)	Gynecologic examination, pap smear, pelvic ultrasound

In phase I, all participants underwent extensive clinical, laboratory and environmental exposure measurements aimed at identifying clinical, biological, environmental and genetic factors associated with SHS. In the second phase, a long-term yearly clinical follow-up will be performed until 2024, with the purpose of better understanding how SHS, environmental and genetic risk factors contribute to the development of major chronic diseases.

We estimated the sample size based on the incidence of cerebrovascular and cardiovascular morbidity in the peripheral arterial disease study (PERART/ARTPER) [26]. The incidence of cerebrovascular and cardiovascular morbidity was reported to be 1124 and 2117 per 100,000 person-year respectively in a Mediterranean low cardiovascular risk population [26]. Based on the combined incidence in ARTPER cohort (3241 per 100,000 population), *α* = 0.05, *β* = 0.10, proposed odds ratio (OR) of 1.50 (high SHS score group vs. low SHS score group), and the prevalence ratio of high SHS score group vs. low SHS score group of 1:5, the sample sizes of high and low SHS score groups were estimated to be 278 and 1390 respectively (PASS 11) [27]. Considering attrition rate of 10 %, a sample size of 306 for high SHS group and 1530 for low SHS group met the minimum required sample size. In actuality, 9078 participants were recruited, which is 5 times that of the required sample size.

#### Inclusion criteria

All adults (from 18 to 65 years old) participating in the baseline investigation, and those who were also willing to be involved in future follow-up investigations were included into the study.

#### Exclusion criteria

Participants currently suffering from diabetes (self-reported diabetes or FPG ≥ 7.0 mmol/L at the investigation), hypertension (self-reported hypertension, or SBP ≥ 140 mmHg, or DBP ≥ 90 mmHg at the investigation), hyperlipemia (self-reported), cardiovascular or cerebrovascular conditions (including self-reported atrial fibrillation, atrial flutter, heart-failure, myocardial infarction, transient ischemic attack, and stroke), any type of cancer (self-reported), and gout (self-reported) were excluded.

### Phase I cross-sectional survey

#### Data collection by questionnaires

All participants were asked to complete a set of combined self-administered questionnaires (including SHSQ-25) with the assistance of a well-trained research assistant. The questionnaires collected the following information:

##### SHS measurements

SHS questionnaire (SHSQ-25) was used to measure SHS [12–16]. The SHSQ-25 contains 25 items under the 5 domains of fatigue, the cardiovascular system, the digestive tract, the immune system, and mental status. Each subject was asked to rate a specific statement on a 5-point scale. The raw scores of 1–5 on the questionnaire were recorded as 0–4. SHS scores were calculated for each respondent by summing the ratings for the 25 items. A high score (≥35) represents a high level of SHS (poor health), with a score of ≥35 regarded as SHS, and the remains are ideal health [28]. The Cronbach’s *α* coefficient of the SHSQ-25 in previous investigation was 0.91, indicating good individual internal consistency [12].

##### Demographics

Age (date of birth), sex, marital status, nationality, education level, and household income.

##### Lifestyle, physical activity and environmental factors

Information on drinking history, active smoking or passive smoking at home or work was recorded. The current information on tobacco, alcohol and tea consumption, dietary intakes of meat, fruit, vegetable, dairy, cereals and salt were also collected.

Physical activity and sedentary behavior were assessed using the short form of the International physical activity questionnaire (IPAQ) [29]. We collected the average sleeping hours over a 24-h period.

##### Medical history and physical symptoms

Current use of medication and supplements were collected, as well as medical history including age at diagnosis of the following diseases;

Cardiovascular or cerebrovascular conditions: atrial fibrillation, heart-failure, myocardial infarction, transient ischemic attack, and stroke.

Endocrine conditions: diabetes and gout.

Neurological conditions: Alzheimer’s disease, vascular dementia (multi-infarct dementia), Parkinson’s disease, attention deficit (hyperactivity) disorder, anxiety disorder.

Sleep disorders: narcolepsy and obstructive sleep apnoea.

Other medical conditions: cancer, and cataclasis.

Sleep symptoms such as day-time somnolence, snoring frequency, witnessed apnoeas, frequency of unrefreshed sleep and waking tired or falling asleep while driving were assessed using the Epworth sleepiness scale [30] and the Berlin questionnaire [31].

Mental health was also assessed using the depression, anxiety and stress scale (DASS21) [32] and depressive severity measured using the patient health questionnaire-9 (PHQ-9) [33]. Information on current treatment for depression including medication, exercise or psychological counseling was also collected.

#### Physical examination

##### Blood sample collection and biochemistry tests

Blood samples were collected from the antecubital vein of all participants in the morning under fasting conditions. They were stored in vacuum tubes containing EDTA (ethylene diamine tetraacetic acid) and coagulation tubes. A range of haematological and biochemistry tests (Table 2) were conducted on fresh samples at the central laboratory of the Staff Hospital of *Jidong* oil-field of Chinese National Petroleum. Fasting blood glucose was measured with the hexokinase/glucose-6-phosphate dehydrogenase method. Cholesterol and triglyceride concentrations were determined by enzymatic methods (Mind Bioengineering Co. Ltd, Shanghai, China). Blood samples were also measured using an auto-analyzer (Hitachi 747; Hitachi, Tokyo, Japan) at the central laboratory of the Staff Hospital of *Jidong* oil-field of Chinese National Petroleum. For all participants, serum creatinine, cholesterol, high-density lipoproteins (HDL-C), low-density lipoproteins (LDL-C), triglycerides and glucose levels were assessed. In subgroup analysis studies, various biomarkers of blood cells, serum and plasma were measured: C-reactive protein, homocysteine, estrogens, androgens, vitamin D, lipoprotein-associated phospholipase A2 (Lp-PLA2), insulin, and glycosylated hemoglobin HbA1c.

**Table 2:** Haematology, biochemistry and biological specimen banking in the COACS

	Analysate
Red blood cells	Haemoglobin
	Red corpuscle count
	Haematocrit
	Mean corpuscular volume
	Mean corpuscular
	Haemoglobin concentration
	Red blood cell distribution width
White blood cells	White cell count Total count
	Differential count
Platelets	Platelets Count
	Mean platelet volume
Urea	Urine specific gravity
	Ery
	Urea nitrogen
	Uric acid (UA)
	Creatinine (Cr)
	Urine protein
Liver function tests (plasma)	Alkaline phosphatise
	Alanine transaminase (ALT)
	Aspartate aminotransferase (AST)
	Phosphatise Transglutaminase (TG)
Liver function tests (serum)	HBsAg
	Anti-HBs
	HBeAg
	Anti-HBe
	Anti-HBc
Lipids (plasma)	Total cholesterol (TC)
	Total bilirubin (TBIL)
	Triglycerides (TG)
	Low density lipoprotein (LDL)
	Very Low density lipoprotein (VLDL)
General chemistry (plasma)	C-reactive protein
	Homocysteine
	Steroids
	Glucose
	Insulin
	Glycosylated hemoglobin
Bio-specimen banking	
White blood cells	DNA, RNA extraction and analyses
Serum	Pedtidome profiling
Plasma	Glycome

Blood samples were processed and separated onsite for biospecimen banking (−80 °C). DNA and RNA were extracted and stored in the laboratory of Beijing Key Laboratory of Clinical Epidemiology, Beijing, China.

##### Cardiovascular and cerebrovascular

A resting 12-lead electrocardiogram (ECG) and rhythm strip was recorded digitally using a Cardio Perfect PC-Based resting ECG system (Welch Allyn). The ankle brachial index (ABI) measurement was used to determine peripheral arterial disease (PAD) using a standard method [34]. Transcranial Doppler was performed by two experienced neurologists with portable examination devices (EME Companion, Nicolet, Madison, WI, USA) to determine intracranial arterial stenosis (ICAS), which was diagnosed according to the peak flow velocity based on published criteria [35]. Bilateral carotid duplex ultrasound was used to evaluate extracranial carotid stenosis (ECAS), with carotid stenosis (≥50 %) based on recommendations from the Society of Radiologists in Ultrasound Consensus Conference [36].

##### Respiratory

Forced expired volume in one second (FEV_1_) and forced vital capacity (FVC), before and 15 min after salbutamol (200 mcg) delivered via a metered-dose inhaler and spacer, were measured using an Asyone™ spirometer and compared with predicted values [37].

##### Cognition

Memory and attention were assessed using the Cognitive Drug Research (CDR) computerized assessment system (United BioSource Corporation, UK), which is widely used in clinical and longitudinal studies, including the dementias, and had been shown to be sensitive to subtle cognitive changes [38].

##### Anthropometry and body composition

Standing height, waist and hip girth, and weight were measured with the participant lightly-clothed and shoeless using standard anthropometric techniques. Body mass index (BMI) was calculated as well as waist to hip ratio (WHR). Blood pressure was determined to the nearest 2 mmHg using a mercury sphygmomanometer with a cuff of appropriate size. Two readings of systolic blood pressure (SBP) and diastolic blood pressure (DBP) were taken at a five-minute interval, and the mean of the two readings was taken as the BP value. Arterial hypertension was defined based on the following information alone or in combination: (1) with a history of arterial hypertension; (2) using antihypertensive medication; or (3) a systolic blood pressure >140 mmHg, or a diastolic blood pressure of >90 mmHg.

Dual energy X-ray absorptiometry (DEXA) scans (AP spine and dual femur) were undertaken to assess bone mineral density (BMD) using a GE Lunar Prodigy Pro densitometer and enCORE Version 13 (GE Health) software. Bone mineral density was measured in grams per centimeter squared (g/cm^2^) and young adult T-scores and age-matched z-scores were derived using the combined Geelong/Lunar reference database (GE Health).

### Phase II Scheduled follow-up study

#### Follow-up

The study participants will be followed up via face-to-face interviews once every year in a routine medical examination up to December 31, 2024, or up to the occurrence of a final event as defined in the study, or occurrence of death. In every interview, information on SHS, demographics, lifestyle, activity and environment, medical history and physical symptoms, blood samples, anthropometry and body composition, cardiovascular and cerebrovascular, and cognition will be collected. Data on clinical outcomes will be collected through a standard operational procedure follow-up system. The follow-up system involves linkage of the study base to files from general practitioners in the study area and subsequent collection of information from letters of medical specialists and discharge reports in case of hospitalization. With respect to the vital status of participants, information will also be obtained regularly from the municipal health authority in *Tangshan* city.

A diagnosis of major disease is confirmed only after review of the medical records by an End Points Committee of physicians that includes experts such as cardiologists, neurologists, and oncologists. An End Points Committee of physicians including membership, role and responsibilities has been approved by the Project Executive Committee.

#### Primary outcomes

##### Cardiovascular events

Clinical cardiovascular outcomes will be coded by study physicians and medical experts in the field according to the International classification of diseases, 10th edition (ICD-10). Incident coronary heart disease is defined as the occurrence of a fatal or nonfatal myocardial infarction (I21), other forms of acute (I24) or chronic ischemic (I25) heart disease, sudden (cardiac) death (I46 and R96), death caused by ventricular fibrillation (I49), or death resulting from congestive heart failure (I50) during follow-up [39]. Other outcomes include heart failure [40] and atrial fibrillation [41].

##### Cerebrovascular events

The primary outcome will be the first occurrence of stroke, either the first nonfatal stroke event, or stroke death without a preceding nonfatal event. A nonfatal stroke is defined as a focal neurological deficit of sudden onset and vascular mechanism that lasts for >24 h. Cases of fatal stroke will be documented by evidence of a cerebrovascular mechanism obtained from all available sources, including death certificates, medical insurance and hospital records. Stroke will be classified according to the criteria as ischemic stroke, and hemorrhagic stroke (ICD-10 codes: G45, I63, I61, I60). The diagnosis will be confirmed by the evidences of brain X-ray computed tomography (CT) or magnetic resonance imaging (MRI) [42], which are classified into brain infarction, intracerebral hemorrhage, and subarachnoid hemorrhage. Lacunar infarction and stroke diagnosed just by imaging or as the second diagnosis will be excluded.

#### The secondary outcomes

The secondary outcomes will include: type 2 diabetes (T2D), chronic obstructive pulmonary disease (COPD), and other chronic diseases.

Type 2 diabetes (ICD-10: E11) is defined as the presence of any of the following criteria: (1) fasting plasma glucose value of ≥126 mg/dl (7.0 mmol/L) on two occasions or symptoms of diabetes and a casual plasma glucose value of ≥200 mg/dl (11.1 mmol/L) or both, (2) current use of insulin or oral hypoglycemic agents, or (3) a positive response to the question:“Has a doctor ever told you that you have diabetes?”

COPD (ICD-10: J40-J47) is defined by a moderate-to-severe obstructive spirometry (FEV_1_/FVC < 0.70 and FEV_1_ < 80 % predicted), and/or as COPD diagnosed by a specialist in internal medicine (mainly respiratory physicians or internists with a subspecialty in respiratory medicine) based upon the combination of clinical history, physical examination and spirometry. Probable COPD is defined by a mild obstructive spirometry (FEV_1_/FVC < 0.70 and FEV_1_ ≥ 80 % predicted) and/or as COPD diagnosed by a physician in another medical specialty (e.g., a general practitioner). Clinical outcomes will be collected during our continuous follow-up and include respiratory and non-respiratory death, hospitalizations due to exacerbations of COPD as well as moderate to severe COPD exacerbations treated with systemic corticosteroids and/or antibiotics.

Information about other chronic diseases, including hypertension, Alzheimer’s disease, Parkinson’s disease and other neurodegenerative diseases, cancer, chronic hepatitis, chronic osteoarticular diseases, osteoporosis, and chronic kidney disease, will also be collected.

### Data capture and management

Hard copies of questionnaires were scanned and converted to electronic portable document file (PDF) format and data was extracted and verified on-site using Cardiff TeleForm software (Verity Inc. Sunnyvale, CA). These data, along with automatic data capture capabilities from most of the devices being used are stored with the existing COACS collection which is managed by Beijing Key Laboratory of Clinical Epidemiology, Beijing, China.

### Quality control (QC)

Each participant was assisted by a well-trained research assistant to fill in the questionnaires, and will be followed up by face-to-face interviews once every year in a routine medical examination. All research assistants, interviewers and physical examiners are trained in all items of the questionnaires, or all aspects of measurements (using standardized techniques). Trainings are conducted on-site, and within the laboratories of each of the participating investigators under the supervision of experienced staff, until the required standard of testing and competency has been achieved.

During the course of the survey, regular central monitoring is also undertaken to assess the distribution of certain key variables, the time delay with blood processing and consistency of the data collected. On-site monitoring visits are undertaken every 6-month by staff from Staff Hospital of *Jidong* oil-field of Chinese National Petroleum. In addition, QC monitoring regarding the follow-up has also been conducted by staff from a third party (Recovery Medical Technology Development Corporation).

### Ethics statement

This study is performed according to the guidelines of the Declaration of Helsinki [43]. Approvals have been obtained from Ethical Committees of the Staff Hospital of *Jidong* oil-field of Chinese National Petroleum, Beijing Tiantan Hospital, and Capital Medical University. These approvals will be renewed every 5 years. Written informed consent has also been obtained from each of the participants.

### Statistical analyses

#### Baseline cross-sectional study

Questionnaire results and the results of physical and cognitive testing were recorded to calculate and compare the level of risk factors in participants with SHS or ideal health. Normality distributions of continuous variables were tested by the Kolmogorov–Smirnov tests. Continuous variables were represented as Mean ± Standard Deviation, or Median (Percentile 25th–75th), while discrete variables were represented as numbers (proportion). The differences between groups were tested by *t* test or Wilcoxon rank sum test (skewed continuous variables or graded variables), or Chi square test (discrete variables). All reported *P* values were two-sided, and *P* < 0.05 was considered statistically significant.

#### Follow-up longitudinal analysis

Changes from baseline to yearly follow-up in risk factors, sociodemographic factors, and the primary or secondary outcomes will be measured and relationships between them will be investigated using survival analysis, logistic regression, linear regression and cox regression models, or standard longitudinal data regression methods (such as generalized mixed models and generalized estimation equation). These analyses will be focused on an understanding of the patterns and processes of chronic diseases and on the identification of factors that are contributing to the onset and incidence of chronic diseases.

## Cross-sectional survey results/baseline characteristics

**Fig. 1:**
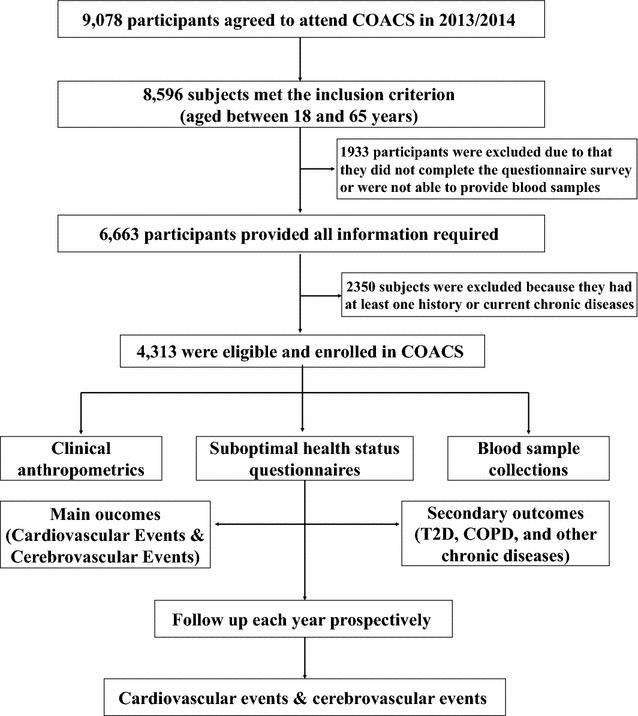
Flowchart of the China suboptimal health cohort study (COACS). *T2D* type 2 diabetes, *COPD* chronic obstructive pulmonary disease

The study recruited 9078 participants from *Caofeidian* district. However, 4765 were excluded from the study for one or more of the following reasons: they did not meet the inclusion criteria; did not complete the questionnaire; were unable to provide a blood sample, or had either a current, or a history of, chronic disease. Therefore, a total of 4313 participants were included in the COACS study, with 389 of SHS (SHS score ≥35) and 3924 of ideal health (SHS score <35). This would result in a power of 93.3 % in a planned 4-year follow-up or power of 90.90 %, given 10 % of withdraw rate (see Fig. 1).

The descriptive characteristics of participants in COACS are summarized in Table 3. The mean age of the participants was 36.9 (±10.5) years with 53.8 % being women. The majority (59.1 %) had a household income between Chinese Yuan (CNY) ¥3000–5000 per month, and 87.6 % of them were married. About 70.9 % of participants had completed college school or higher. Most of the participants never smoked (75.3 %), never drank (70.2 %), were Chinese Han (96.8 %), and had normal BMI (57.1 %). About 50.3 % of participants were active in physical activity, and 52.6 % had medium salt intakes. The prevalence of SHS in the investigated population was 9.0 %, higher in women than in men (10.6 vs. 7.2 % in male and female, respectively).

**Table 3:** Baseline demographic characteristics of the COACS population stratified by gender

Characteristics	Total (n = 4313)	Women (n = 2319)	Men (n = 1994)	*P* value
Age (years)*	36.9 ± 10.5	37.5 ± 10.4	36.2 ± 10.6	<0.001^#^
Nationality				
Han	4176 (96.8 %)	2249 (97.0 %)	1927 (96.6 %)	0.524
Others	137 (3.2 %)	70 (3.0 %)	67 (3.4 %)	
Socioeconomic status^a^				
≤¥3000	1393 (32.8 %)	820 (35.9 %)	573 (35.9 %)	<0.001^#^
¥3001–5000	2509 (59.1 %)	1316 (57.5 %)	1193 (57.5 %)	
>¥5000	343 (8.1 %)	151 (6.6 %)	192 (6.6 %)	
Marital status				
Married with spouse	3778 (87.6 %)	2087 (90.0 %)	1416 (84.8 %)	<0.001^#^
Widowed, separated, or divorced	69 (1.6 %)	47 (2.0 %)	18 (1.1 %)	
Never married	466 (10.8 %)	185 (8.0 %)	277 (14.1 %)	
Highest education completed				
Illiteracy or compulsory education	431 (10.0 %)	255 (11.0 %)	176 (8.8 %)	<0.001^#^
High school	823 (19.1 %)	510 (22.0 %)	313 (15.7 %)	
College school or higher	3059 (70.9 %)	1554 (67.0 %)	1505 (75.5 %)	
Smoking history				
Never	3247 (75.3 %)	2291 (98.8 %)	956 (47.9 %)	<0.001^#^
Current	976 (22.6 %)	28 (1.2 %)	948 (47.5 %)	
Former	90 (2.1 %)	0 (0.0 %)	90 (4.5 %)	
Drinking history				
Never	3024 (70.2 %)	2203 (95.1 %)	821 (41.2 %)	<0.001^#^
Moderate	762 (17.7 %)	60 (2.6 %)	702 (35.2 %)	
Heavy	524 (12.2 %)	54 (2.3 %)	470 (23.6 %)	
Body mass index (kg/m^2^)^b^				
<18.5	124 (3.1 %)	101 (4.6 %)	23 (1.3 %)	<0.001^#^
18.5–23.9	2284 (57.1 %)	1484 (67.9 %)	800 (44.1 %)	
24.0–27.9	1244 (31.1 %)	485 (22.2 %)	759 (41.8 %)	
>28.0	349 (8.7 %)	115 (5.3 %)	234 (12.9 %)	
Physical activity				
Inactive	1430 (34.1 %)	848 (37.5 %)	582 (30.1 %)	<0.001^#^
Moderately	655 (15.6 %)	383 (16.9 %)	272 (14.0 %)	
Very active	2114 (50.3 %)	1032 (45.6 %)	1082 (55.9 %)	
Salt intake				
Low	891 (20.7 %)	5794 (25.0 %)	312 (15.6 %)	<0.001^#^
Medium	2268 (52.6 %)	1243 (53.6 %)	1025 (51.4 %)	
High	1154 (26.8 %)	497 (21.4 %)	657 (32.9 %)	
Blood Pressure (mmHg)				
Systolic blood pressure*	116.9 ± 10.8	114.1 ± 10.9	120.1 ± 9.7	<0.001^#^
Diastolic blood pressure*	74.5 ± 8.2	72.1 ± 8.2	77.2 ± 7.3	<0.001^#^
Fasting blood glucose (mmol/L)*	4.93 ± 0.45	4.88 ± 0.43	4.99 ± 0.46	<0.001^#^
Total cholesterol (mmol/L)*	4.26 ± 0.82	4.22 ± 0.82	4.31 ± 0.82	<0.001^#^
Triglycerides (mmol/L)*	1.32 ± 1.02	1.09 ± 0.66	1.59 ± 1.26	<0.001^#^
Suboptimal health status				
SHS (SHSQ score ≥35)	389 (9.0 %)	246 (10.6 %)	143 (7.2 %)	<0.001^#^
Ideal health (SHSQ score < 35)	3924 (91.0 %)	2073 (89.4 %)	1851 (92.8 %)	

Continuous variables were represented as mean ± standard deviation, or median ± interquartile range, while discrete variables were represented as number (proportion)

^a^80 subjects provided missing data in variable of ‘Socioeconomic status’

^b^282 subjects provided missing data in variable of ‘Body Mass Index’

* Mean ± standard deviation

^#^ *P* < 0.05

The gender, socio-economic status, marital status, highest education completed, physical activity, salt intake, blood pressure and triglycerides differed significantly between the SHS group and ideal health group (*P* < 0.05), whereas the differences of age, ethnicity, smoking, drinking, BMI, fasting blood glucose and total cholesterol were of no statistical significance (Table 4).

**Table 4:** Factors distribution in participant with or without Suboptimal Health Status

	Total, n = 4313 (n, %)	SHS, n = 389 (n, %)	Ideal health, n = 3924 (n, %)	*P* value
Gender				
Male	1994 (46.2 %)	143 (36.8 %)	1851 (47.2 %)	<0.001^#^
Female	2319 (53.8 %)	246 (63.2 %)	2073 (52.8 %)	
Age (years)*	36.9 ± 10.5	36.4 ± 9.1	37.0 ± 10.7	0.287
Nationality				
Han	4176 (96.8 %)	375 (96.5 %)	3801 (96.9 %)	0.352
Others	137 (3.2 %)	14 (3.6 %)	123 (3.1 %)	
Socioeconomic status^a^				
<¥3000	1393 (32.8 %)	148 (38.8 %)	1245 (32.2 %)	0.008^#^
¥3000–5000	2509 (59.1 %)	208 (54.6 %)	2301 (59.5 %)	
>¥5000	343 (8.1 %)	25 (6.6 %)	318 (8.2 %)	
Marital status				
Married with spouse	3778 (87.6 %)	349 (89.7 %)	3429 (87.4 %)	<0.001^#^
Widowed, separated, or divorced	69 (1.6 %)	16 (4.1 %)	53 (1.4 %)	
Never married	466 (10.8 %)	24 (6.2 %)	442 (11.3 %)	
Highest education completed				
Illiteracy or compulsory education	431 (10.0 %)	28 (7.2 %)	403 (10.3 %)	0.032^#^
High school	823 (19.1 %)	68 (17.5 %)	755 (19.2 %)	
College school or higher	3059 (70.9 %)	293 (75.3 %)	2766 (70.5 %)	
Smoking history				
Never	3247 (75.3 %)	296 (76.1 %)	2951 (75.2 %)	0.926
Current	976 (22.6 %)	85 (21.9 %)	891 (22.7 %)	
Former	90 (2.1 %)	8 (2.1 %)	82 (2.1 %)	
Drinking history				
Never	3024 (70.2 %)	264 (67.9 %)	2760 (70.4 %)	0.485
Moderate	762 (17.7 %)	71 (18.3 %)	691 (17.6 %)	
Heavy	524 (12.2 %)	54 (13.9 %)	470 (12.0 %)	
Body mass index (kg/m^2^)^b^				
<18.5	124 (3.1 %)	22 (5.9 %)	102 (2.8 %)	0.163
18.5–23.9	2284 (57.1 %)	210 (56.8 %)	2074 (57.1 %)	
24.0–27.9	1244 (31.1 %)	102 (27.6 %)	1142 (31.5 %)	
>28.0	349 (8.7 %)	36 (9.7 %)	313 (8.6 %)	
Physical activity				
Inactive	1430 (34.1 %)	166 (44.0 %)	1264 (33.1 %)	0.001^#^
Moderately	655 (15.6 %)	64 (17.0 %)	591 (15.5 %)	
Very active	2114 (50.3 %)	147 (39.0 %)	1967 (51.5 %)	
Salt intakes				
Low	891 (20.7 %)	79 (20.3 %)	812 (20.7 %)	0.024^#^
Medium	2268 (52.6 %)	179 (46.0 %)	2089 (53.2 %)	
High	1154 (26.8 %)	131 (33.7 %)	1023 (26.1 %)	
Blood pressure (mmHg)				
Systolic blood pressure	116.9 ± 10.8	114.4 ± 11.1	117.1 ± 10.7	<0.001^#^
Diastolic blood pressure	74.5 ± 8.2	73.1 ± 8.3	74.6 ± 8.2	0.001^#^
Fasting blood glucose (mmol/L)*	4.93 ± 0.45	4.90 ± 0.46	4.94 ± 0.45	0.161
Total cholesterol (mmol/L)*	4.26 ± 0.82	4.22 ± 0.78	4.27 ± 0.82	0.271
Triglycerides (mmol/L)*	1.32 ± 1.01	1.21 ± 0.74	1.33 ± 1.04	0.005^#^

^a^80 subjects provided missing data in variable of ‘Socioeconomic status’

^b^282 subjects provided missing data in variable of ‘Body Mass Index’

* Mean ± standard deviation

^#^ *P* < 0.05

## Discussion

We define a subclinical, reversible stage of pre-chronic disease as the SHS [11, 12, 14]. It is a physical state between health and disease, characterized by the perception of health complaints, general weakness, chronic fatigue and low energy levels within a period of 3 months [11, 12]. We have also developed a tool to measure SHS. Our SHSQ-25 assesses five components of health: fatigue, mental health, the digestive system, the cardiovascular system and the immune system [12–16]. However, whether and how the SHS contribute to the main chronic diseases (such as cardiovascular and cerebrovascular events, T2D, and COPD) remains unclear, and no existing cohort is available to investigate these contributions. To our knowledge, this is the first cohort study that includes measurements of SHS, which will enable the thorough characterization of SHS, and precisely estimate the incidence of chronic disease. This is based on the comprehensive assessments of both subjective and objective health statuses, together with lifestyle and environmental factors.

At baseline of COACS, we found that gender, age, smoking, BMI, salt intake and blood pressure levels were significantly associated with SHS. Females had a higher SHS rate than males (10.6 vs. 7.2 %), indicating that women have a higher risk of developing NCD. The imbalance of socio-economic, marital and education statuses between genders may contribute to this phenomenon, along with the natural physiological differences between males and females [16].

In addition, middle socio-economic status (¥3000–5000 of household income) appears to be a protective factor for SHS, suggesting that middle income in a community is associated with better health status. However, the existence of high physical activities in this group may be a confounding factor, thus providing a rational explanation to this outcome. We also found that marital status (widowed, separated, divorced) is a risk factor for suboptimal health status. SHS has also been observed to be related to less physical activity and higher salt intakes (>6 g per day), which are known risk factors of cardiovascular and cerebrovascular diseases [14, 44]. The blood pressure and triglycerides were slightly lower in subjects of SHS than those of health (114.4 ± 11.1 vs. 117.1 ± 10.7 mmHg for SDP, 73.1 ± 8.3 vs. 74.6 ± 8.2 mmHg for DBP, and 1.21 ± 0.74 vs. 1.33 ± 1.04 mmol/L for triglycerides). It is prudent to note that blood pressure and triglycerides levels that might be caused by the subjects of hypertension or hyperlipidemia had been excluded from the recruitment.

Whilst SHS has been showed to be associated with cardiovascular risk factors and chronic psychosocial stress [14, 16], little is known as to whether SHS contributes independently to the incidence of NCD. The SHSQ-25 is a multidimensional, self-report symptom inventory including 5 health domains (fatigue, the cardiovascular system, the digestive tract, the immune system, and mental status), which match well with the physiological, psychological and social dimensions [12, 13] corresponding to the greater understanding of WHO’s definition of health. SHS is associated with cardiovascular risk factors (higher SBP, DBP, FBG, total cholesterol, and lower HDL cholesterol) and contributes to the development of cardiovascular diseases [14]. In addition, significantly higher levels of plasma cortisol and GRb/GRa mRNA ratio were observed in the high SHS group than these in low SHS group [16]. The mechanism underlying SHS has yet to be ascertained, and the objective measurements for SHS are currently under investigation [16]. The COACS study was designed to examine the prevalence and association of SHS in a general population, and to evaluate prospectively the relationship between SHS and risk factors contributing to the incidence of NCD. This was achieved by using our novel SHSQ-25, along with objective measurements of biomarkers. Preventable NCD accounts for an estimated 80 % of deaths and 70 % of disability-adjusted life-years lost in China [7]. Therefore, a multidimensional and multidisciplinary health promotion and disease management plan of NCD are urgently needed. The preventive and predictive approach, including community-based strategies and interventions for high risk factors at a population level, rely on a comprehensive understanding of relevant, current and integrated data on the prevalence, clustering of disease, known risk factors, and discovery of new risk factors [45]. The COACS will add novel knowledge across a broad range of areas by:

### Stratifying the participants resident in a real community environment into SHS and ideal health

Using our SHSQ-25, the participants in the cohort are categorized into SHS and ideal health groups. To our knowledge, this is the first attempt in investigating whether, and to what extent, SHS contributes to the incidence of chronic disease. If so, an unprecedented opportunity would then exist for the early detection or intervention of chronic disease.

### Repeated measurement of biomarkers promote precise predication of disease progression

Continuous collection of multiple biomarkers, together with banking of biological samples (serum, plasma, DNA and RNA) will facilitate future investigations of both known and potential new factors that put health at risk. The collection will be an important resource for future genetic studies. This is especially so considering the value of well-characterized populations for collaborative genetic disease mapping. The collection will also allow future genetic and functional studies to examine pathological pathways in disease processes at the genome, transcriptome, proteome, metabolome and glycome levels. The combination of objective biomarkers at Omics levels, together with subjective health measures such as the SHSQ-25, will produce optimal and precise prevention and prediction of disease progression at an individual’s suboptimal health stage.

## Conclusion

In summary, 4313 participants (53.8 % women) aged 18–65 years were included in the cohort, and the prevalence of SHS in all participants was 9.0 % using a threshold of SHS score of 35. Based on the baseline cross-sectional study, the pilot study showed that risk factors for chronic diseases (such as socio-economic status, marital status, highest education completed, physical activity, salt intake, the blood pressure and triglycerides)differed significantly between subjects of SHS (SHS score ≥35) and those of ideal health (SHS score <35). The COACS study is a community-based, real-life environment, prospective study to investigate whether SHS, along with life-style and other socio-economic factors, contributes to the incidence of chronic disease in Chinese adults. Furthermore, the COACS study affords the opportunity to longitudinally analyze the genetic, lifestyle and environmental factors that may determine onset and etiology of targeted chronic disease. The study together with the designed prospective cohort provides a chance to characterize and evaluate the effect of SHS systemically, and it thus generates an unprecedented opportunity for the early detection and prevention of chronic disease.

## Abbreviations

SHS: suboptimal health status
COACS: China suboptimal health cohort study
SHSQ-25: suboptimal health status questionnaire-25
PERART/ARTPER: peripheral arterial disease study
IPAQ: International physical activity questionnaire
DASS21: depression, anxiety and stress scale
PHQ-9: patient health questionnaire-9
EDTA: ethylene diamine tetraacetic acid
HDL-C: high-density lipoproteins
LDL-C: low-density lipoproteins
Lp-PLA2: lipoprotein-associated phospholipase A2
BMI: body mass index
WHR: waist to hip ratio
SBP: systolic blood pressure
DBP: diastolic blood pressure
DEXA: dual energy X-ray absorptiometry
BMD: bone mineral density
ECG: electrocardiogram
ABI: ankle brachial index
PAD: peripheral arterial disease
ICAS: intracranial arterial stenosis
ECAS: extracranial carotid stenosis
FEV_1_: forced expired volume in one second
FVC: forced vital capacity
CDR: cognitive drug research
ICD-10: International classification of diseases, 10th edition
CT: X-ray computed tomography
MRI: magnetic resonance imaging
COPD: chronic obstructive pulmonary disease
PDF: portable document file
QC: quality control
WHO: World Health Organization
